# Pericytes on placental capillaries in terminal villi preferentially cover endothelial junctions in regions furthest away from the trophoblast

**DOI:** 10.1016/j.placenta.2020.10.032

**Published:** 2021-01-15

**Authors:** Shelley E. Harris, Kate SH. Matthews, Eleni Palaiologou, Stanimir A. Tashev, Emma M. Lofthouse, Jennifer Pearson-Farr, Patricia Goggin, David S. Chatelet, David A. Johnston, Maaike SA. Jongen, Anton M. Page, Jane K. Cleal, Rohan M. Lewis

**Affiliations:** a Human Development and Health, Faculty of Medicine, University of Southampton, UK; b Biomedical Imaging Unit, Faculty of Medicine, University of Southampton, UK; c Institute for Life Sciences, University of Southampton, UK

**Keywords:** Three-dimensional imaging, Microvasculature, Placenta, Pericytes

## Abstract

**Introduction:**

Pericytes are a common feature in the placental microvasculature but their roles are not well understood. Pericytes may provide physical or endocrine support for endothelium and in some tissues mediate vasoconstriction.

**Methods:**

This study uses serial block-face scanning electron microscopy (SBFSEM) to generate three-dimensional (3D) reconstructions of placental pericytes of the terminal villi and transmission electron microscopy (TEM) to study pericyte endothelial cell interactions. The proportion of endothelial cell junctions covered by pericytes was determined.

**Results:**

The detailed 3D models of placental pericytes show pericyte structure at a new level of detail. Placental pericytes have many fingers extending from the cell body which can span multiple capillary branches. The proportion of endothelial cell-cell junctions covered by pericytes was significantly higher than pericyte coverage of capillary endothelium as a whole (endothelium: 14%, junctions: 43%, p < 0.0001). However, the proportion of endothelial cell-cell junctions covered by pericytes in regions adjacent to trophoblast was reduced compared to regions >3 μm away from trophoblast (27% vs 62% respectively, p < 0.001). No junctional complexes were observed connecting pericytes and endothelial cells but there were regions of cell membrane with features suggestive of intercellular adhesions.

**Discussion:**

These data suggest that the localisation of pericytes on the villous capillary is not random but organised in relation to both endothelial junctions and the location of adjacent trophoblast. This further suggests that pericyte coverage may favour capillary permeability in regions that are most important for exchange, but limit capillary permeability in other regions.

## Highlights

• Three-dimensional imaging highlights the structure of placental pericytes.
• Placental pericytes preferentially cover endothelial junctions.
• The proportion of covered junctions decreased in regions adjacent to trophoblasts.
• The localisation of placental pericytes suggests endothelial coverage is non-random.
• Junction coverage may alter capillary permeability in key regions of exchange.

## 1 Introduction

Placental function underpins optimal fetal development and health across the life course. The placenta is a heterogeneous organ containing multiple cell types, however, most of these are poorly understood. Pericytes are one such cell type which may play an important role in placental vascular function.

Placental villi are tree-like structures containing fetal blood vessels and stroma, which are surrounded by syncytiotrophoblast, to form the interface between the fetal and maternal blood. Recent studies have shown that the volume of villi with smooth muscle α-actin positive perivascular cells, correlates positively with term placental weight and thickness [1], suggesting there is a functional role for the stromal parts of the villous tree. The terminal villi, at the tips of the villous branches, are the primary sites of solute exchange from maternal blood. Before reaching the capillary, maternal nutrients are transported across the placental syncytiotrophoblast, which forms the primary barrier between the mother and the fetus, and released into the connective tissue separating the trophoblast from the fetal capillaries. Passive permeability of hydrophilic solutes across the placental capillary endothelium is believed to be mediated via the presence of para-cellular routes, primarily endothelial cell-cell junctions [2,3].

The permeability of the endothelial junctions is a key component determining the level of passive diffusion across the capillary lining [4,5]. Tight junctions sit between these endothelial cells and anchor them together, maintaining their strength and integrity [6,7], thus potentially affecting the rate of transfer through the placental barrier [8]. Endothelial cells respond to changes in physical stresses by dynamic remodelling, and the location of endothelial junctions reflects the position of these forces [9].

Pericytes are located on the abluminal capillary side of the endothelium and consist of a central cell body with multiple fingers that grip the capillaries [10,11]. They appear to have functional significance in several different tissues, such as the kidney, brain and heart [[11], [12], [13]]; however, the role of pericytes in the placenta is unclear. Previous studies in the kidney have suggested that pericytes influence vascular tone in medullary blood flow autoregulation through oxygen and sodium sensitivity [14]. Additionally, *in vitro* co-culture experiments with rat brain capillary endothelial cells and rat brain pericytes has suggested that pericytes decrease the permeability of the endothelial cells via secretion of transforming growth factor-β1 [15]. It has also been proposed that pericytes can have a mechanical role in modulating the vascular tone; smooth muscle α-actin has been detected within pericytes in renal medulla [16].

Pericytes may play a key role in placental development and homeostasis. The proportion of vessels surrounded by pericytes increases as gestation advances, indicating a potential role in vascular development [17]. Pericyte foot processes have been located encircling capillaries suggesting a role for pericytes in regulating vessel contraction [17]. Pericytes are known to express pro-angiogeneic factors, such as, vascular endothelial growth factor (VEGF), transforming growth factor beta (TGF-β) and angiopoietins, suggesting they are associated with the development of new capillaries [[18], [19], [20]]. The platelet-derived growth factor B (PDGF-B) is also involved in placental angiogenesis and can also play a role in pericyte proliferation and recruitment [21]. In mice, the absence of signalling between PDGF-B and its receptor (PDGFRβ) in the placenta can decrease pericyte number and coverage along the capillary, which in turn can increase capillary diameter and permeability [19,22], suggesting pericytes play an important role in capillary formation and function. Pericyte localisation in the bovine lung capillary [23] and rat cremaster muscle [24] has been shown to be non-random with preferential coverage of the junctions, but this has not been studied in the placenta [25].

This study sought to construct three-dimensional (3D) models of human placental tissue to investigate the relationships between pericytes and capillary endothelial cells from terminal villi, to better understand their role within placenta microvasculature. Investigation of the coverage of endothelial junctions by pericyte fingers will elucidate whether pericytes can influence passive diffusion or aid in maintaining vascular tone.

## 2 Methods

### 2.1 Samples

This study used term human placental tissue, collected after delivery from uncomplicated pregnancies with written informed consent and ethical approval from the Southampton and Southwest Hampshire Local Ethics Committee (11/SC/0529). All methods and experiments were approved and performed in accordance with the relevant guidelines from the Southampton and Southwest Hampshire Local Ethics Committee and the University of Southampton.

### 2.2 Wholemount confocal imaging

To visualise the spatial arrangement of pericytes, wholemount confocal microscopy was performed, as previously described [26]. Briefly, placental tissue was collected and fixed overnight in 4% formaldehyde in phosphate-buffered saline (PBS). Prior to labelling the samples were washed in PBS and cut into 2–3 mm^3^ blocks and permeabilised in 1% Triton-X-100 (Sigma, UK). Samples were incubated in 2% bovine serum albumin (Fisher Scientific, UK) to block non-specific binding before incubating with smooth muscle α-actin (1:10,000, Sigma, UK) and PDGFRβ (20 μg/ml, Santa Cruz Biotechnologies, Heidelberg, Germany) antibodies overnight at 4 °C. Endothelial cells were visualised using fluorescein labelled *Aleuria aurantia* Lectin (AAL; 10 μg/ml, Vector Labs, UK) suspended in PBS, in the absence of calcium and magnesium. Samples were cleared through an increasing series of thiodiethanol (TDE, Sigma, UK) and then imaged on a 4-channel confocal laser scanning microscope (SP5, Leica, UK). Stacks of 40–60 images were generated using sequential imaging with 800 × 800 pixels with a z-axis spacing of 1 μm [26].

### 2.3 SBFSEM processing and imaging

Samples from six placentas were collected as soon as possible after delivery, and small pieces (no bigger than 2 mm^3^) fixed in 3% glutaraldehyde in 0.1 M cacodylate buffer at pH 7.4 until processing for SBFSEM. Processing of samples has been described in detail elsewhere [26,27]. Blocks were imaged using a Gatan 3view (Gatan, Abingdon, UK) inside an FEI Quanta 250 FEGSEM (ThermoFisher, Eindhoven, NL) at 3.0 kV accelerating voltage, spot size 3 and with a vacuum level of 40 Pa. Between 400 and 2000 images were collected per stack, ranging from 25 to 50 nm in thickness.

Stacks from three out of the six placentas were processed using the image analysis software Fiji [NIH, USA; http://rsb.info.nih.gov/ij; [28] using a Gaussian blur filter (sigma radius 2) and enhanced contrast (0.1% saturated pixels). The region of interest in the first slice of each stack was selected to include a complete pericyte and endothelial cell. On each slice in the relevant stacks, the pericyte regions were identified and labelled (segmented) using the software Avizo 9.3.0 (ThermoFisher, Eindhoven, NL) to allow 3D reconstruction. A skeletonised pericyte was generated in Avizo and the Fiji plugin Skeletonize3D [29] was used for subsequent analysis of the skeleton junction and branch number.

### 2.4 TEM processing and imaging

TEM processing and imaging is described in detail elsewhere [3,26]. Briefly, fixed placental fragments were washed in cacodylate buffer before placing in 2% osmium tetroxide in 0.1 M sodium cacodylate. Samples were infiltrated with 2% aqueous uranyl acetate and dehydrated using a graded ethanol series. Specimens were then treated with 50:50 Agar low viscosity (ALV) resin:acetonitrile overnight followed by fresh ALV resin for 6 h. Finally, specimens were embedded in fresh ALV resin and polymerised for 16 h at 60 °C. Gold/silver ultrathin sections (90 nm) were cut, stained with Reynolds lead stain and imaged on a Tecnai 12 TEM (ThermoFisher, Eindhoven, NL) using an EMSIS Morada camera (EMSIS, Muenster, Germany). The distance between pericytes and endothelial cells was estimated in TEM images from five placentas using Fiji.

### 2.5 Quantification of pericyte junction coverage

To estimate the proportion of endothelial junctions covered by pericytes, the image stacks generated from SBFSEM were systematically sampled to select every 50th image from a random starting image. A total of sixteen stacks were used from six placentas. Stacks were excluded if the selected images did not contain endothelial junctions or if they were not of sufficient quality to provide clear determination of whether the pericytes were covering the junctions. A total of 142 images were quantified. The total number of endothelial junctions present on the image were counted alongside the number of junctions covered by a pericyte (cell body or process). Only junctions where the pericyte was fully covering the junction were counted; a fully covered function was defined as the width of the junction being covered by a pericyte, rather than touching the junction at one side. The proportion of endothelial surface area covered by pericytes was determined using a stereological approach as described previously [3]. To determine the proportion of covered junctions near the trophoblast versus those not, the number of pericyte-covered junctions was counted in relation to their location within the villi. Pericytes were readily identifiable as cells in direct contact with an underlying endothelial cell and which closely followed the contour of the endothelial cell.

### 2.6 Data analysis and statistics

Data were expressed as mean and standard error of the mean (SE). Statistical analysis was conducted using Prism (Graphpad, US). In the first instance, normality was determined using a Shapiro-Wilk test. For data normally distributed, a paired student's t-test was used to determine statistical difference; data which failed normality was assessed using a Mann-Whitney test. The observed distribution of the percentage of pericytes covering junctions was analysed by Fisher's exact test and their distribution of location was analysed by one-way ANOVA followed by a Tukey test. Significance was accepted as p < 0.05.

## 3 Results

### 3.1 Confocal imaging of villi

**Fig. 1:**
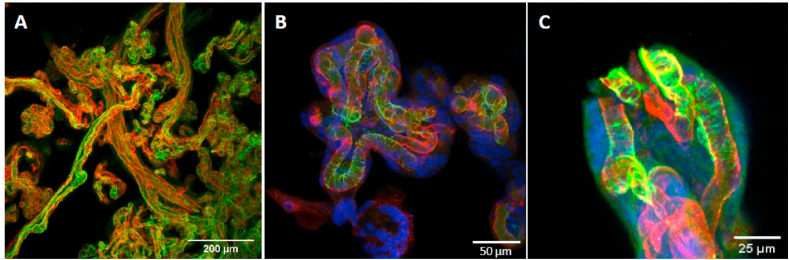
Confocal images of placental pericyte fingers stained with smooth muscle α-actin and PDGFRβ (note n = 1). (A) Low resolution image showing staining with smooth muscle α-actin (green) is widespread across the villi (endothelium = red). (B) High resolution image showing pericyte fingers expressing smooth muscle α-actin (green) around vessels (AAL = red, DAPI = blue). (C) High resolution image of pericyte bodies and fingers expressing PDGFRβ (green), AAL (red) and DAPI (blue).

Staining with smooth muscle α-actin demonstrates the widespread presence of pericytes in terminal villi (Fig. 1a). The identification of these cells as pericytes was confirmed by the presence of fingers around the capillaries (Fig. 1b). PDGFRβ staining was possible in one sample which clearly showed pericyte fingers around the capillary in the same way as the smooth muscle α-actin (Fig. 1c).

### 3.2 Reconstruction of pericytes by SBFSEM

**Fig. 2:**
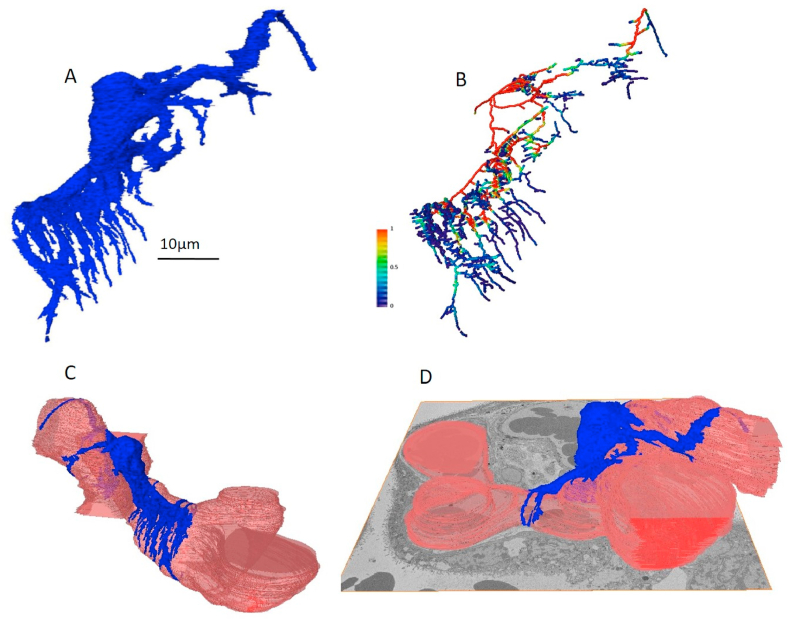
Pericytes have a close relationship with the capillary endothelium. (A) Segmentation of a whole placental pericyte (blue) including visualisation of the pericyte fingers extending from a cell body. (B) A skeletonised representation of the pericyte showing extensive branching (coloured by thickness). (C) The fingers appear not to fully encircle the capillaries and can span multiple capillary branches from one cell body (D). An animation of the pericyte being rotated can be seen in supplementary material.

A whole pericyte was reconstructed from 1249 serial images (depth: 62.5 μm; Fig. 2a). The 3D reconstruction highlights the extent to which the pericyte fingers grip the capillary; the three main limbs span more than one capillary branch and then branch further, with 75 junctions resulting from 32 limbs extending from the original cell body. The pericyte cell bodies tended to be positioned on the capillary side furthest away from the syncytiotrophoblast. While there was considerable variation, typically a pericyte finger extended laterally from the cell body and with two bifurcating digits at the distal end and two lateral digits budding from a more proximal position (Fig. 2b). Skeletonisation of the pericyte indicates that the pericyte fingers are thinner compared to the cell body (Fig. 2b). The reconstruction confirmed the current knowledge of pericytes consisting of a central cell body with fingers extending around the capillary typically not more than half a capillary diameter (Fig. 2c and d; supporting Video S1).

**Fig. 3:**
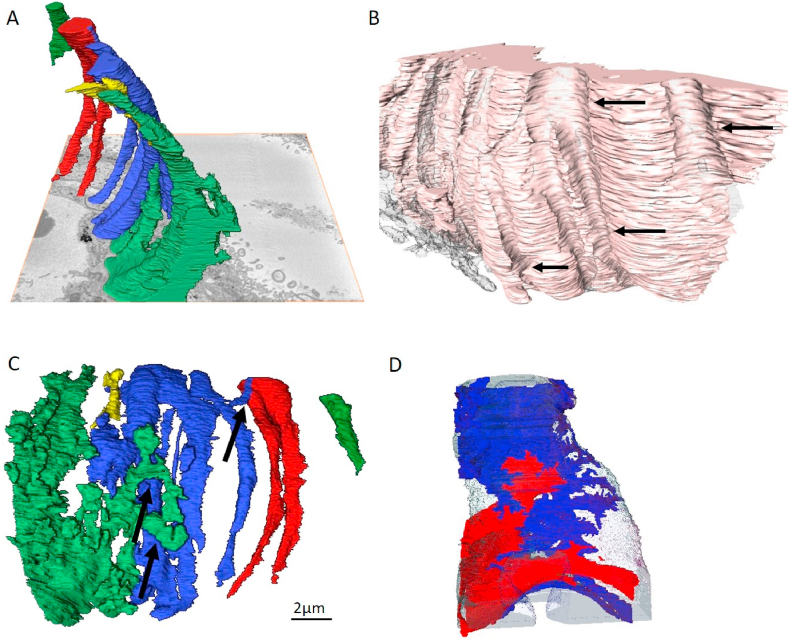
High-resolution 3D reconstruction of pericyte finger tips. (A) Fingers bend around the capillary away from the syncytiotrophoblast and (B) appear to leave indents on the endothelium of the capillary wall (pericytes are transparent. Indents indicated by arrows). (C) Pericyte fingers branch and in some cases overlap (arrows). (D) Two separate pericytes intertwine (one blue and the other red). The individual colours denote separate and discrete entities.

To further investigate the structure of the pericyte fingers and their relationship to the endothelium, 3D reconstructions were generated from two high-resolution stacks from two placentas containing 366 and 399 serial images. In one stack, fingers appear to bend around the capillary, parallel to each other (Fig. 3a) and appear to grip into the endothelium, leaving indentations (Fig. 3b). Interestingly, the pericyte fingers do not fully cover the capillary but are only located on the side furthest away from the syncytiotrophoblast under the cell body. The fingers appear to become thinner as they move closer to the trophoblast layer. The reconstruction also shows how in this pericyte two fingers overlap each other (Fig. 3c, supporting Video S2). A second stack of images shows two different pericytes combining to encircle the capillary (Fig.3d); a finger from one pericyte can be seen to extend between the fingers of another separate pericyte.

### 3.3 Quantification of pericyte junction coverage

**Fig. 4:**
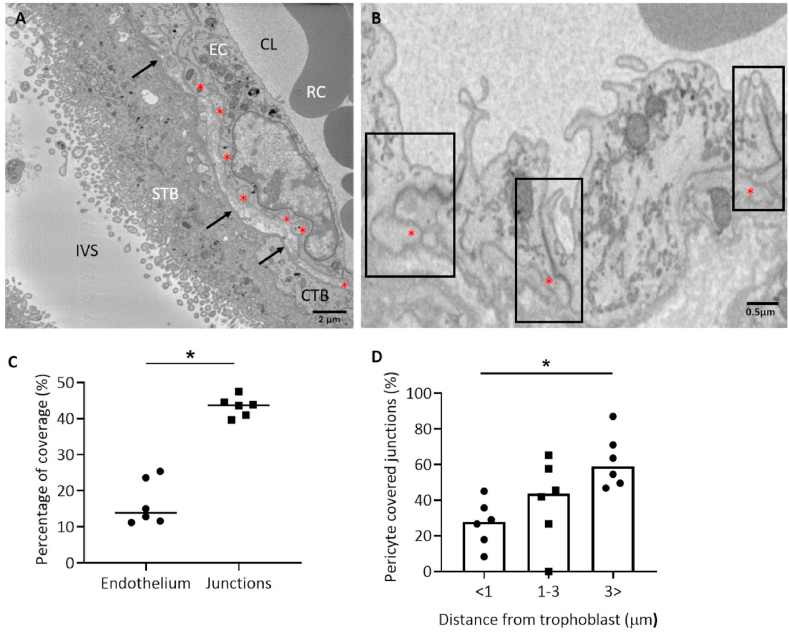
(A) SBFSEM image of terminal villus. Pericyte fingers (*) sit flush with the endothelial surface and there is an absence of junctional complexes attaching them to endothelial cells. The image shows the capillary lumen (CL) containing a red blood cell (RC), the surrounding endothelial cells (EC), the syncytiotrophoblast (STB), cytotrophoblast (CTB) and the intervillous space (IVS). Arrows indicate the location of the capillary basal lamina. (B) Pericytes (*) located covering endothelial tight junctions (black boxes); (C) pericyte coverage of endothelial junctions was higher than the proportion of endothelial surface area coverage. *significantly different from percentage of pericytes covering endothelial surface area, analysed by paired student's t-test and Fisher's exact test, p ≤ 0.0001, n = 6 placentas. (D) Percentage of pericyte covered junctions at differing distances from trophoblasts. *Significantly different from <1 and 1–3 μm groups, analysed by one-way ANOVA followed by a post hoc Tukey test, p ≤ 0.001.

From individual SBFSEM images, pericyte fingers appeared to be embedded within the endothelium to create a smooth contiguous surface (Fig. 4a). The proportion of endothelial junctions covered by a pericyte (43.4 ± 1.1%) was more than double the proportion of pericytes covering endothelial surface area (16.6 ± 2.6%, p < 0.0001, n = 6 placentas, Fig. 4b and c) as reported in our previous paper [3]. When the junctions were grouped by distance from trophoblast, those junctions more than 3 μm away from trophoblast had pericyte coverage of 62.1 ± 6.2%, junctions between 1 and 3 μm away from trophoblast had pericyte coverage of 39.5 ± 9.6% and junctions within 1 μm of trophoblast had pericyte coverage of 27.1 ± 5.3% which was significantly lower than for those junctions more than 3 μm away from the trophoblast (p < 0.001, n = 6 placentas, Fig. 4d). The proportion of junctions >3, 1–3 and <1 μm away from trophoblast were 41%, 17% and 42%, respectively out of 982 junctions.

### 3.4 Pericyte endothelial cell junctions

**Fig. 5:**
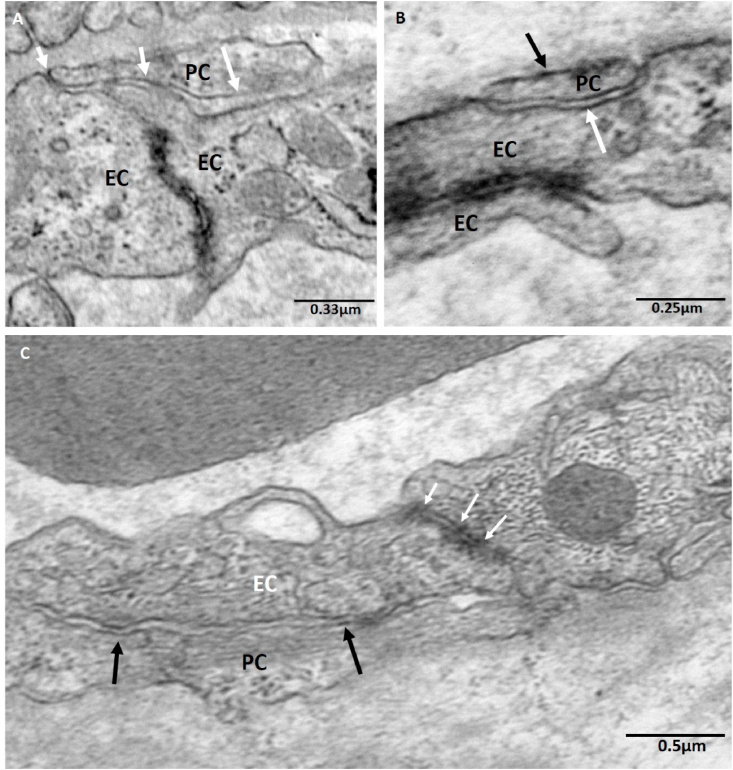
TEM images illustrating the association between pericyte fingers (PC) and the abluminal surface of the endothelium (EC). (A) The junctions in the endothelial cell-cell junction can be clearly observed. Potential non-junctional connections are illustrated by white arrows. (B) The capillary basal laminal is also visible (black arrow). (C) Sections of membrane were observed with higher regions of electron density (black arrows). Pericyte-endothelial cell junctions appeared looser in comparison to endothelial cell-cell junctions which contain complexes which appear to be tight junctions as indicated by the white arrows.

No example of apparent junctional complexes between pericytes and endothelial cells was observed in the TEM images at the resolutions available (Fig. 5a and b). However, sections of adjacent pericyte and endothelial cell plasma membrane were observed with regions of higher electron density which may be suggestive of looser forms of adhesion (Fig. 5c). Compared to endothelial cell-cell junctions, which contain abundant junctional complexes, the pericyte-endothelial cell junction was typically looser in appearance and had a membrane-membrane spacing of 35 ± 1 nm (n = 22 junctions from five placentas).

The capillary basal lamina could be observed in many TEM images overlying the endothelial cells and the pericytes (Fig. 5b). The capillary basal lamina is generally less apparent than the trophoblast basal lamina and does not show clearly in SBFSEM images.

## 4 Discussion

This study presents the first 3D reconstructions of pericytes based on ultrastructural images from human placental terminal villus capillaries. A key observation of this study was that pericytes preferentially cover endothelial cell-cell junctions, but that this coverage is halved in regions adjacent to the trophoblast where most transfer will occur. As covering endothelial cell-cell junctions is likely to affect capillary permeability, it is possible that pericytes may be acting to regulate capillary permeability in a manner that enhances the efficiency of placental transfer.

Overall, the pericyte cell body has numerous fingers that typically bifurcate at the distal end and often have smaller lateral branches. The fingers of one pericyte may grip onto multiple capillary branches, but do not fully encircle the vessel; the longest fingers extended approximately half a capillary circumference. In many cases, the pericyte fingers were embedded in the endothelium so that the surface of the endothelium and pericytes formed a continuous common surface, suggesting that the pericyte fingers may have constricted into the endothelium or that their position is held in place as the endothelium was pushed out by internal hydrostatic forces. Previous evidence suggests that venous constriction within the placenta regulates the fetomaternal water flux [30]; agonists that alter vascular tone are likely to affect water exchange and this could be, in part, mediated by capillary pericytes. The closeness and spatial arrangement of the fingers needs further investigation. In some cases, interlinking of pericyte fingers was observed which may provide additional support around the capillary.

Coverage of endothelial cell-cell junctions by pericytes would increase the diffusive distance, which would be expected to reduce diffusion through the junction and so reduce capillary permeability. This has previously been reported in rodent lung and muscle in response to inflammation [25]. The observation that there is preferential coverage of endothelial junctions by pericyte fingers in the placenta of a non-complicated pregnancy is somewhat unexpected. However, when this was investigated in relation to the position of the trophoblast, rather than on the capillary as a whole, a different picture emerged. Compared to regions greater than 3 μm away from the trophoblast, regions adjacent to trophoblast had reduced pericyte coverage of endothelial cell-cell junctions. Assuming that pericyte coverage does reduce diffusion through endothelial junctions, this arrangement is likely to favour diffusion in and out of the capillary in regions adjacent to the trophoblast and reduce it in others. This could both facilitate transfer of nutrients into the fetal circulation but also direct fetal wastes to regions where they will be cleared most rapidly.

The relatively high proportion of junctions observed within 1 μm of the trophoblast does suggest that there may be a higher proportion of junctions in this region. However, without measuring the lengths of endothelium in each region this cannot be concluded from this study and further work is required.

The extent to which pericytes act as a diffusional barrier when they cover endothelial cell-cell junctions will depend on how tight that association is and how much it increases the diffusive path length. The TEM imaging suggested that there was a relatively loose association when compared to endothelial cell-cell junctions. No junctional complexes were observed between the two cell types, although there were regions of electron dense membrane visible on the TEM which are suggestive of a looser form of cadherin based connection [31]. However, immunohistochemical studies are required to demonstrate the nature of these connections. This suggests that the main effect of the pericyte on diffusion will be due to an increase in diffusive path length. The preference for junction coverage may also indicate a potential role for pericytes in maintaining structural integrity of these junctions. By their nature, endothelial junctions are structural weaknesses in the capillary endothelium [6], and therefore pericytes may help strengthen these regions. A more rigorous investigation into the distance and coverage of junctions would be needed to understand the relationships between to two parameters. However, this is beyond the scope of this study.

In contrast to villous capillary pericytes, intermediate villous arteriole and venule pericytes appear to cover a greater proportion of the endothelial surface [3]. It is likely that pericytes in different regions of the microvasculature have different phenotypes and functions [3,25]. Pericyte populations within the vasculature are known to be variable within different tissues, probably reflecting hydrostatic pressures [25]. Higher proportions of pericytes in distal regions suggests a pressure driven mechanical role for pericytes in protection of vessel walls [25]. However, it remains to be determined whether these differentially located pericytes represent distinct cell populations. Markers for smooth muscle actin have previously been used to classify centrally located contractile villus from more peripheral non-contractile villi; in humans, the perivascular stromal compartment of the most peripheral branch of the villus is free of γ-smooth muscle actin immunoreactivity [1].

Manual segmentation of SBFSEM images and reconstruction of 3D images is time consuming. As a consequence of this, the number of cells that have been studied is limited. The development of machine learning approaches may allow more rapid reconstruction of cells from SBFSEM stacks, thereby opening the way to more quantitative approaches [32].

In conclusion, this study provides a detailed 3D ultrastructural reconstruction of a pericyte and suggests a role for pericytes in regulating capillary permeability. Additional investigations into pericytes and their relationship with placental microvasculature structure and permeability may further our understanding of their role in uncomplicated and complicated pregnancies.

## Declaration of competing interest

The authors have no conflicts of interest.
